# Current state and recent developments of child psychiatry in China

**DOI:** 10.1186/s13034-015-0040-0

**Published:** 2015-05-13

**Authors:** Yi Zheng, Xixi Zheng

**Affiliations:** Beijing Anding Hospital, Capital Medical University, 100088 Beijing, PR China; The Chinese Society of Child and Adolescent Psychiatry, 100088 Beijing, PR China; Beijing Institute for Brain Disorders, 100069 Beijing, PR China; Peking Union Medical College Hospital, No.1 Shuaifuyuan Dongcheng, 100730 Beijing, PR China

**Keywords:** Child mental health, Culture, China, Psychiatry

## Abstract

China has a population of 1.3 billion, of which 238 million are children under age 15. The rapid economic development and social reforms that have taken place in recent years all had a great influence on child and adolescent mental health. Though a nationwide prevalence study for child and adolescent mental disorders in China is lacking, several regional studies have shown the prevalence of mental disorders in children to be close to the worldwide prevalence of 20%. This article reviews the current status of Chinese child psychiatry, the prevalence of specific disorders in China and the influence of culture on the diagnosis and treatment of child and adolescent mental disorders. Several important social issues are also explored in detail, including the one child policy and left-behind children of migrating workers. Changes in family structures along with the growing competitions in life have weakened the traditional social support system. As a result childhood behavioral problems, mood disorders in young college students, substance abuse and youth suicide are all increasing in China. Many who suffer from mental disorders are not adequately cared for because the scarcity of qualified service providers and pathways to care. This article also lists some challenges and possible solutions, including the multidisciplinary and culture sensitive service model for child mental health. Relevant laws, policies and regulations are also introduced.

China has a large population of children. The social reforms that have taken place in recent years and the rapid economic development have had a great influence on child and adolescent mental health. Increasing social stress, the growing migration of workers and the one child policy have changed the traditional family structures and social support systems. This review aims to provide an up-to-date description of child and adolescent psychiatry in China focusing on how this young subspecialty faces the challenges of contemporary Chinese society.

## Prevalence of child mental disorders

China has a population of 1.3 billion; of which 238 million are children under 15 years old [1]. Though a nation-wide prevalence study is lacking, some regional epidemiological studies show that the prevalence of mental disorders in children is close to the worldwide prevalence of 20% (See Table 1) [2-6]. Studies from different time periods demonstrate an increasing trend in the overall prevalence of child mental disorders. The preliminary results of a nationwide epidemiological study suggest that 15% of Chinese children suffer from mental health problems and the prevalence of some disorders, such as anxiety disorders, are increasing [7].

**Table 1:** **Prevalence of child mental disorders in selected regions of China**

**Authors**	**Time**	**Cities**	**Age, years**	**Sample size**	**Tools**	**Prevalence**
Wang YF, Chen YC et al.	1988	Beijing	7-14	2432	CBQ	8.3%
Xi RE, Xu TY et al.	1992	22 cities	4-16	24013	CBCL	12.97%
Yang ZW, Li XR et al.	1997	Hunan	4-16	8644	CBCL, DMS-IV	14.89%
Tang GZ, Guo LT et al.	2005	Chengdu	11-18	1740	CBCL	15.1%
Guan BQ, Luo XR et al.	2009	Hunan	5-17	9495	DSM-IV	16.22%

CBQ: Children Behavior Questionnaire, Rutter; CBCL: Child Behavior Checklist, Achenbach; DSM-IV: Diagnostic and Statistical Manual of Mental Disorder-IV, American Psychiatric Association.

There are regional epidemiological studies for some specific childhood mental disorders, such as autism spectrum disorders (ASD), attention deficit hyperactivity disorders (ADHD) and Tourette disorder (TD).

### Autism spectrum disorder (ASD)

ASD is a relatively new disorder in China, with the first few cases reported by Guotai Tao in 1986 [8]. Because of the low prevalence of ASD, a large population has to be surveyed when conducting prevalence studies. The Chinese versions of the Clancy Autism Behavior Scale (CABS) which was available in Chinese in the late 90s has been widely used in epidemiological studies of ASD [9]. Table 2 summarized some major studies on the prevalence of ASD in China [10-18].

**Table 2:** **Studies on prevalence of autism in China**

**Time**	**Region**	**Definition**	**Age (years)**	**Screening (diagnose) instrument**	**Sample size**	**Prevalence (per 10,000)**	**Gender ratio (M: F)**	**Urban/rural ratio**
2000	Fujian	ASD	0-14	ABC (CCMD-2, DSM-III-R)	10802	2.80	1.77 (P > 0.05)	0.50 (P > 0.05)
2002	Changzhou	ASD	2-6	CABS (CARS, CCMD-2-R)	3978	17.89	2.00	N/A
2003	Zunyi, Guizhou	ASD	3-12	ABC (DSM-IV)	10412	5.76	N/A	N/A
2004	Beijing	PDD	2-6	CABS (CARS, DSM-IV)	21866	15.30	1.08 (P > 0.05)	1.38 (P > 0.05)
2004	Tianjin	ASD	2-6	CABS (CARS, DSM-IV)	7316	11.00	6.00 (P < 0.05)	1.75 (P > 0.05)
2006	National based survey	ASD associated disability	0-17	Screen for disability first (ICD-10)	77301	2.38	2.09 (P < 0.05)	1.03 (P > 0.05)
2010	Tianjin	ASD	1.5-3	CHAT (CARS, DSM-IV)	8428	26.60	4.15 (P < 0.05)	0.61 (P > 0.05)
2014	Changchun	ASD	0-6	ABC (CARS, CCMD-3)	9714	15.44	N/A	N/A

A meta-analysis of 18 studies showed the pooled prevalence of childhood autism to be 11.8 per 10,000 individuals (95% confidence interval (CI): 8.2, 15.3) in Mainland China and 26.6 per 10,000 (95% CI: 18.5, 34.6) in Mainland, Hong Kong and Taiwan [19]. This is lower than the prevalence rate of 6-10‰ for ASD reported in developed countries [20,21]. In 2006 the second survey of disabled people included ASD children [22]. In this survey, the prevalence of ASD in children aged 0–6 years is 11 per 10,000. Of which 36.9% are disabled according to WHO International Classification of Functioning, Disability, and Health (WHO-ICF) [23]. ASD is more prevalent in boys than in girls, but ethnicity, social economic levels have no effect on the prevalence of this disorder.

Some speculations have been made as for why China has a relatively low prevalence of ASD. First, the methodology of prevalence studies can affect results. Analysis of these studies shows that the prevalence of ASD are most strongly associated with the choice of screening instrument [19]. Most studies in China used CABS as the screening instrument and Childhood Autism Rating Scale (CARS) as the diagnostic tool. This may be related to the wider availability of the Chinese version of CABS, which is a 14-item instrument developed in the 1969 with little revision and update in recent years [24]. The administration of CABS takes less time than other instruments such as Autism Behavior Checklist (ABC). But, studies have shown a weaker consistency of CABS with the diagnostic criteria in DSM-IV [25]. Additionally, in most studies the children who had negative screen results were not given a diagnostic assessment, which can also lead to under diagnosis of ASDs. The age group of the studies can also affect the results; most studies in China were done in the 2–6 years age group while in developed countries the trend was toward early recognition and screening and the concept of adult autism is also been increasingly accepted [26]. Secondly, the awareness of ASD among the public is an important factor in epidemiological studies since parents or other caregivers are the one who filled out the screening and diagnostic questionnaires. Chinese parents, in particular, are reported to face higher parenting stress and stigma with autistic children and experience more internalization and self-blame [27]. This may explain the unwillingness to identify autistic children among Chinese parents.

### Attention Deficit Hyperactive Disorder (ADHD)

The prevalence studies of Attention Deficit Hyperactivity Disorder (ADHD) in China began in the early 1980s. Since then, more than 30 studies put the prevalence of ADHD between 0.73% and 14.8%. Table 3 summarized some epidemiological studies [28-34] highlighting their screening and diagnosing criteria and the prevalence of each subtype of ADHD as defined in the Diagnostic and Statistical Manual of Mental Disorders (DSM).

**Table 3:** **Selected studies on prevalence of ADHD in China**

**Time**	**Region**	**Definition**	**Age (years)**	**Screening (Diagnostic) Instrument**	**Sample size**	Prevalence (%)*	**Age group with the highest prevalence (years)**	**Gender ratio (M: F)**	**Other risk factors**
1981	Beijing	ADD	6-13	Self made questionnaire (ICD-9)	2770	5.8 (N/A)	9	7 (P < 0.05)	Lower educational level of the parents
1983	Hebei	ADD	6-13	Self made questionnaire (DSM-III)	1588	3.3 (N/A)	N/A	4.8 (P < 0.05)	N/A
2003	Guilin	ADHD	5-12	Conners (DSM-IV)	9162	4.25 (C 1.44, I 1.00, HI 1.81)	8-9	2.18 (P < 0.05)	Birth injury, Lower educational level of parents
2007	6 cities in Northeast	ADHD	6-12	Self made questionnaire (DSM-IV)	1051	5.4 (C 1.14, I 0.67, HI 3.6)	9	1.6	No different between city and rural areas. Lower education level of parents
2009	Shanghai	ADHD	5-15	19 item questionnaire (DSM-IV)	5648	4.6 (C 1.8, I 2.4, HI 0.4)	6-7	2.41 (P < 0.05)	N/A
2010	Shenzhen	ADHD	7-13	Conners PSQ and TRS (DSM-IV)	8193	5.39 (C 3.73, I 1.21, HI 0.45)	5-6	2.94 (P < 0.05)	N/A
2011	Sichuan	ADHD	6-16	19 item questionnaire (DSM-IV)	2350	4.81 (C 1.40, I 2.64, HI 0.77)	6-7	2.53 (P < 0.05)	Positive family history, Birth injury, Less parental care
2014	Xinjiang	ADHD	6-14	Conners PSQ (DSM-IV)	2066	4.7%(C 1.54, I 2.42, HI 0.73)	N/A	2.03 (P < 0.05)	N/A

*(Subtype, C = combined, I = Inattentive, HI = Hyperactivity).

A Meta analysis [35] pooled the prevalence data from 33 studies done in China from 1980 to 2011 and found the prevalence of ADHD to be increasing over the years, from 3.7% in 1980–1989 to 4.3% in 1990–1999 and 6.2% in 2000–2011 (P < 0.05). The most important factor affecting prevalence rate is the diagnostic instrument used, with the highest prevalence rate in studies using DSM-IV, the lowest in studies using Chinese Classification of Mental Disorders (CCMD). But the overall prevalence in China (5.7%) is slightly higher than the worldwide-pooled prevalence of 5.29% [36]. Some important moderators for prevalence includes the diagnostic criteria used, the method used in screening ADHD symptoms and the incorporation of functional impairment as part of the definition of ADHD. Some researchers believe that since no subjective diagnostic method exists for ADHD, the objective evaluation of the rater plays an important role in diagnosis. Cultural difference between China and western countries may result in inter-rater differences [37].

### Tourette syndrome

Tourette syndrome (TS) is introduced to China in the early 1980s. The worldwide prevalence of TS is around 1% [38]. A study done in 1983 screened 17727 children and diagnosed 43 cases of TS. The reported prevalence of TS in China is 0.24% with higher prevalence in urban areas [39]. More recent epidemiological study of 9742 school-aged children in Wenzhou [40] showed a prevalence of 0.43% in with no significant difference between urban and rural areas. Study of children between 6 and 16 years in Beijing showed similar prevalence for Tourette disorder (TD) (2.26% for TD, 0.47% for TS). This means that at least 2 million children in China are suffering from this condition. The male to female ratio is between 5–8:1 [41]. The diagnosis of TS is made clinically with no subjective tests to help confirm the diagnosis. Affected children usually suppress the tic in public places and clinics, this is specially so in China where children are expected to behave themselves in public. This posed a cultural problem in epidemiological studies and may lead to an underestimation of the true prevalence of this condition.

## Diagnosis and treatment for child mental disorders

### Diagnosis of mental disorders

The diagnosis of mental disorder is different from that of most other medical conditions. It relies on subjective reporting of symptoms and the level of functional impairment. In the field of child psychiatry, problems that parents or teachers perceive as being serious and warranting attention are shaped by prevailing cultural beliefs and values. Thus the recognition of certain symptoms and the labeling of impairment depend on behavioral norms accepted by a particular culture. A study by Mann et al. [42] compared the ratings of mental health professionals in four different countries including Mainland China on hyperactive-disruptive behaviors. The results indicated that the definition of and attitudes towards hyperactivity are subject to cultural variation. It was found that Chinese and Indonesian clinicians provided higher ratings of hyperactivity than the clinicians from Japan and the United States. In China there is a Chinese diagnostic classification for mental disorders, but the DSM-IV is often used in clinical studies and research. More comparative data and intercultural studies are needed to justify the use of DSM in China and facilitate multicenter international collaboration.

The Chinese Classification of Mental Disorders (CCMD), published by the Chinese Society of Psychiatry, is a clinical guide used in China for the diagnosis of mental disorders. The current version of CCMD-3 was published in 2001. Broad similarities exist between the ICD-10 and CCMD-3. But CCMD-3 also included some variations on the main diagnoses from ICD, and around 40 culturally related diagnoses were added [43]. A survey among 380 psychiatrists in Beijing showed that CCMD-3 is the most commonly used diagnostic system in China (63.8%), followed by the ICD-10 (28.5%) and DSM-IV (7.7%) [44].

Mental disorders for adult and child/adolescent were listed under different categories in CCMD-3. Ten disorders with onset usually occurring in childhood were included in CCMD-3 and were divided into two main categories, namely ‘Mental retardation, and disorders of psychological development with onset usually occurring in childhood and adolescence’ and ‘Hyperkinetic, Conduct, and Emotional disorders with onset usually occurring in childhood and adolescence’. Because of the only-child policy and the family structure in China, the drafting committee of CCMD-3 found that some disorders, e.g. sibling rivalry disorder, scarcely occur in China and the diagnosis would be more aptly called “companion rivalry disorder”[45]. With the release of the new DSM in 2013, Chinese child psychiatrists are trying to update their diagnostic criteria by issuing a series of new guidelines for disorders like ASD and ADHD [46,47].

Clinical assessments are important diagnostic tools for child psychiatrists. The instruments available in China are either translated from English or locally developed. The problem with translated instruments is the norm used in the scoring system is not well established in the culture into which it is translated. Li and colleagues reported that the Child Behavior Checklist (CBCL) and Teacher Rating Form (TRF) were able to distinguish between children with and without ADHD in China [48]. However, use of the U.S. norms and the recommended T score would yield a 50% to 60% false negative rate. Normalization of instrument is often done in regional centers; as a result the application of these instruments in a nationwide scale can be problematic. Liu and colleagues reviewed more than 500 hundred studies on the mental health of Chinese children aging 0 to 6 years [49]. They found that 67.7% of the studies are cross sectional and only one third of the studies are longitudinal. The instrument used in these studies is mostly translated versions of CBCL, Conner’s Children’s Behavior Scale and ABC. However, after 2001, more locally developed instrument started to gain clinical relevance. Some of the well established locally developed instruments include Screening Checklist for Childhood Autism [50], Screening Checklist for Delayed Language development in Age 1-3 [51]. More culturally relevant and locally developed instruments are needed for the screening and treatment monitoring of child and adolescent mental disorders in China.

### The Chinese culture and the help seeking behavioral of patients

In traditional Chinese culture, the mind is in harmony with the body, and the mind-body dichotomy is not widely accepted. In China, many still view mental disorders with disdain. The stigma associated with mental disorder prevented children from expressing their troubled feelings and seeking help. In a study examining help seeking behaviors among different ethnic groups of college students in Hong Kong, Mak et al. find that Chinese Americans and Europeans are more likely to seek help than Hong Kong and Mainland Chinese [52]. A study done in 1993–1994 comparing the help-seeking pattern of Chinese Americans and European Americans found that Chinese people are more likely to turn to non-professionals (relatives, family and pastors) for help [53]. This is validated in another study on suicidal attempts. This study showed that the help-seeking patterns in middle school students with depression and suicidal ideation are mostly turning to friends and parents, with very low levels of professional help-seeking (around 1%). In fact, 30% of students did not seek help at all in face of psychological problems [54].

In a study [37] surveying Chinese and American teachers on the understanding of ADHD, the Chinese samples were more likely to endorse items indicating that ADHD is a reflection of failed parenting or poor effort on the part of the children. The American samples, on the other hand, were less likely to take such a view. This reflects that in Chinese culture mental, illness can be blamed on the family and the individual. A more open and non-judgmental environment should be created for children with mental disorders, especially in China.

### Treatment of mental disorders

Similar to the treatment for mental disorders in developed countries, there is an increased usage of medications in China, perhaps even more so. In the mid to late 1990s, the pharmaceutical industry introduced new psychotropic drugs to the Chinese market. Almost all the psychotropic medications in different therapeutic classes are now available at most tertiary mental health-care centers. Large pharmaceutical companies sponsor most drug-related studies in child psychiatry but randomized double blind controlled trials are still lacking. Compared to adult patients, child and adolescent patients are more likely to receive psychotherapy. Family therapy, group therapy, individual therapy and play therapy are recommended for children and adolescents in China. Cognitive and behavioral therapy and dynamic therapy are also available [32]. For example, for ADHD patients, 77% were treated with central nervous system stimulants, but the proportion of behavioral treatments (either solely on in combination with medications) increased significantly over time [55].

Traditional Chinese medicine (TCM) has been used in treating children with mental disorders. Because the basic diagnostic and treatment philosophy are different in TCM and western medicine, it may be hard to understand the differential diagnostic process of TCM for mental disorders. TCM consider the mind and the body as a functional whole and it views mental disorder as originating from imbalance of the internal organs. Thus, the treatment of mental disorders relies mostly on a psychosomatic approach with the restoration of physiological function and balance as the primary goal. The most widely used methods including acupuncture and TCM medication.

Acupuncture, which involves the use of needles or pressure to specific points on the body, is used widely in TCM and has been used to treat ASD in China. A review included 10 randomized and quasi-randomized controlled trials involving 390 children with ASD. There are no significant differences in the primary outcome measures in the acupuncture group and controlled group, but results suggested acupuncture might be associated with improvement in some aspects of the secondary outcomes of communication and linguistic ability, cognitive function and global functioning [56].

As for TCM medication, there have been little high quality studies on its effect on child mental disorders. However, Chinese researchers are trying to study some TCM medications in stringent randomized controlled trials to assess its efficacy and safety. A recent review analyzed published data on TCM treatment of TS and the result supports a similar efficacy of TCM compared with conventional medication and a superior outcome compared with placebo [57]. A newly developed medication, 5-Ling Granule (5-LGr) (a patented poly-herbal product manufactured from 11 herbal materials) has also undergone a multi-centered, randomized, double blinded, controlled trial with a relative large sample size to treat tic disorder. The result of this trial showed that 5-LGr had similar efficacy in treating tics in Tourette syndrome as Tiapride, a first line tic-suppressing drug used in TS (Zheng et al. in process, trial registration: NCT01501695, detailed herbal name and pharmacological function can be found in the paper).

In China, TCM is a common form of alternative medicine. To some people TCM’s emphasis on harmony and balance between different elements appeal more readily to their notion of a healthy body and mind. And it’s easier for both parents and children to be diagnosed with imbalance of humors than to be labeled with a mental disorder. But in an era of scientific research and evidence-based medicine, TCM has to undergo more rigorous trials to really gain its place in the treatment of mental disorder.

## Problems in the modern Chinese society

### One child policy

The Family Planning Policy, otherwise known as the One Child Policy was introduced in 1979. The Chinese government introduced this policy as a response to the growing social, economic, and environmental issues caused by over-population. The policy, which rewards couples that agree to have just one child, has proved so successful that the birth rate has fallen to only 1.4 children per woman, which is below the replenishment rate (2.1 children per woman) needed to maintain a stable population [58].

However, this successful birth control measure has resulted in new problems, center of which is the problem of an aging population and a skewed sex ratio at birth. From a mental health perspective, the one child policy meant that children do not have to compete with siblings for attention. This could partially explain why overprotection or lack of autonomy was not viewed negatively in most studies with Chinese samples. Another common phenomenon for the only child is the overemphasis on school performance. This is reflected in research showing that while interpersonal conflicts are the primary stressors for “Western” adolescents, poor academic performance prospectively predicts higher levels of depression in Chinese children as young as 8 years of age [59]. In addition, poor academic performance predicts suicidal ideation in Chinese adolescent samples [54]. This could partly be explained by the high expectation families have on the only child.

As the first ‘only child’ generation was born in the 1980s, more and more people are concerned with the way these children were raised. The 4, 2 and 1 family structure is also seen as a potential problem (4 refers to the grandparents, 2 to the parents, and 1 to the child). In 1984, a research was conducted in 6 kindergartens in Beijing with 138 only children and 127 children with siblings focusing on the personality trend of these two groups. The result showed no significant differences in empathic, supportive and aggressive behaviors, but children with siblings scored slightly higher in those domains. Another study lead by Tao et al. studied the impact of one-child policy on child development in 697 preschool children using CBCL [60]. Girls who were only children scored slightly higher on the factors of depression, moody, and temper. Zheng and colleagues conducted several studies on the development of personality and psychological problems of only children. One study of 911 only children in Beijing aged 6 to 12 years showed that the prevalence of social adaption problems was 23% —similar to the average in developed countries [61]. A 6-year multicenter controlled trial of psychosocial development tried to explore the effect of early systemic intervention on psychosocial development in only children. The behavior problems of intervention group were significantly lower than that of control group (P < 0.01). The tendency of psychosocial development, the average IQ, the temperament and the adaptability of intervention group were significantly better than control group (P < 0.05 or 0.01) [62]. This study showed that early systemic intervention benefits the psychosocial development of the only child.

The one child policy is now undergoing a review. Experts are concerned that China’s low birth rate, combined with its aging population, will damage its future economic development. As a result the once strict birth control policy is starting to loosen up. In 2011, if both parents have no siblings, they are allowed to have two children. As of November 2014, the policy also allowed for a family to have two children if either one of the parents have no siblings. As can be expected, the long-term effect of these changes on the psychological wellbeing of children will become a new focus of studies in the coming years.

### Migration workers and left behind children

With the rapid urbanization, the economic gap between cities and rural areas has widened. Rural workforces seek better employment and opportunities in the cities. These often consist of young men and women in their 20s to 40s. Because China‘’s ‘household registration’ system is very rigid, migrated workers are not registered as ‘residents’ in the cities. As a result their children struggle to get services such as education and health service in the cities. Furthermore, rural workers often have lower income, live in more crowded living conditions and cannot afford to bring children with them. That is why the children are often left behind to live in their rural hometowns. This results in the ‘left-behind’ children phenomenon. Left-behind children are defined as children living in their rural home with one or both of their parents working outside their registered resident area [63].

According to a national survey in 2012, the total number of left-behind children has reached 58 million, making up nearly 30% of rural children population [64]. More than half of these left-behind children have both parents working in other cities. A lot of left-behind children (32.67%) are raised by their grandparents. Others (20.70%) are left with other relatives and a small number of them (3.37%) do not have any designated guardian. Compared to 2005, the number of left-behind children in 2012 has grown by 2.4 million. The left-behind children phenomenon and the fast growing number of this special group have raised concerns about their physical and mental wellbeing. Though rural–urban migration is not a phenomenon unique to Chinese society, the scale of migration is unprecedented and the social and economic implications of this phenomenon warrant more attention and research.

In a study assessing the overall quality of life in left-behind children, the mean scores of Pediatric Quality of Life Inventory were lower in the left-behind children than the non-left-behind. While mean physical subscale scores did not differ significantly, the psychosocial summary, emotional functioning, social functioning and school performance scores of left-behind children were lower [65]. Results of the majority of existing studies show that left-behind children are prone to psychological stresses and have more mental health problems. A meta-analysis including 6 controlled studies compared 1465 left behind children and 1401 children in normal family environment. The findings from this and several other studies suggest that left behind children have significantly higher scores in anxiety, loneliness, fear and self-blame [66,67,68]. Other studies found that although no significant differences in the overall mental outcomes between the left behind children and other children existed, certain subgroups of left-behind children were at potential risk [69]. Being raised by grandparents, and going to boarding schools are two independent risk factors for psychological problems while higher education levels of mothers is a protective factor [70]. More psychological problems are seen in boys aged 12–16 years, with oppositional defiant disorder, hyperactivity disorder and poor social interaction being the most troubling problems. A study focused on the left-behind adolescents revealed a higher level of Internet addiction, suicide ideation and thoughts of running away from home along with other social behavioral issues such as smoking and binge drinking [71].

## Current state of Chinese child and adolescent psychiatry: challenges and possible solutions

### Scarcity of child psychiatrists

In China, child psychiatry is a discipline in its nascent stages. Dr. Guotai Tao, the founding father of Chinese child psychiatry, was trained in the USA in 1950s. In 1984, he started the first child psychiatry center in China in Nanjing. Today, in spite of considerable effort, children with mental disorders still lack access to treatment due to the dearth of service providers and a lack of child psychiatrists.

The total number of qualified child psychiatrists in China is less than 500. This small group of doctors certainly cannot provide adequate service for more than 200 million children, and most of these doctors practice in big cities. In China medical students usually receive approximately 20 hours of lecture on clinical psychiatry and practical training in psychiatry wards for approximately two weeks. Child and adolescent psychiatry is hardly taught in medical school. This means that primary care physicians do not have adequate training in child psychiatry. Tertiary care centers usually do not have child psychiatric clinic and even specialized mental hospitals do not have a child psychiatric ward. For children with mental disorders, only 5.8% sought help in a child psychiatric clinic, 9.1% went to pediatrics clinic [72]. Outpatient clinics are the most common form of service for children with mental disorders. A survey done in a mental health center in Shanghai analyzed outpatient data from 1985 to 1999, the result shown that children 6–12 years old are more likely to seek help. But the trend is toward having younger patients (0–3 years). Among the disorders seen in outpatient clinics, ADHD, mental retardation, learning disability and emotional problems are the most common [73].

A multidisciplinary approach could contribute to better service provision. It could take the form of a child and adolescent psychiatrist working with or supervising social workers, or creating positions for social workers within child and adolescent psychiatry departments. In China, traditional social workers are older women from the neighborhoods. But now more colleges and universities are offering degrees for social workers in clinical psychologist and childcare. Also, with the installation of more primary care centers in the community, primary care physicians can play the role of screening and follow-up doctors for children with mental disorders. But more education and training tailored to the need of primary care providers are needed. In order to address this problem, the author is advocating a new form of multilevel collaboration. Pediatricians across the country and primary care physicians are now being trained in early diagnosis and basic treatment for common child mental disorders. They were taught to screen patients for signs of developmental disorders such as ‘Does the three-month-old baby’s eyes follow moving objects?’ or ‘At 18 months, can she make eye contact?’

### The financial burden of mental disorders

Children with mental disorder bring much burden both financially and emotionally to the family. Families of disabled children received more economic assistance than families of normal children. The burden of raising children with disabilities is the highest in children with ASD. Such families have a heavier burden and they need more help in many aspects [74]. Prior to 2005, China’s mental health services were provided in the same manner as all health services in the country. The hospital was the center of the service delivery network and there was little continuity between hospital services and community services. From the beginning of this century, China has invested much in building an effective and functional public health system which was launched as the ‘Central Government Support for the Local Management and Treatment of Severe Mental Illnesses Project’ (also referred to as the ‘686 Project’) [75].

The components of the intervention included patient registration and initial assessment, free medication, regular follow-up in the community, management for community emergencies, and free emergency hospitalization for certain mental disorders. By the end of 2010 a total of 280 000 persons with serious mental disorders had been registered in the system, 200 000 follow-up visits of registered patients had been conducted, free medication was provided 94000 times and free treatment had been provided 12400 times [76].

For other child mental disorders, most are paid by national medical insurance for registered residents of the area. Some children’s medical insurance is covered by their parents’ insurance. Additional commercial medical insurance is also available.

### The mental health law

In 1985, a committee consisted of five senior psychiatrists started to draft a national mental health law. Several key government departments were involved in the process. The draft was revised and released for public consultation only in 2011. Further amendments were made and the Mental Health Law of the People’s Republic of China (referred to as the Mental Health Law below) was finally enacted on May 2013.

Despite its limitations, the Mental Health Law is a great step forward in the protection of psychiatric patients’ civil rights. It aims to promote mental health, improve the quality of mental health services, and protect the human rights of patients with mental disorders during the process of hospital admission, treatment, and discharge. In the newly implemented Mental Health Law, many items are added concerning child mental health. Because China has implemented a nine-year compulsory education program for all school aged children, primary schools have become important functional entity for advocating and improving child mental health and the ideal place to provide related services. Research has shown programs promoting mental health are among the most effective of health promoting school efforts [77]. The mental health law mandates that all levels of school be equipped with psychologists and counseling teachers for mental disorders and psychological problems. Preschool educational institutions must carry out relevant forms of mental health education. In face of traumatic and other stressful events, the school must gather specialists and provide psychological counseling and mental health rescue immediately.

With the implementation of Work Plan for Mental Health in China (2011–2020) [78], China is further promoting the mental health and wellbeing of children and adolescent. The mental health plan requires that by 2015, mental health education in primary school reach 85% of schools in the city and 70% in rural areas. Prevalence of mental disorders should be managed while the awareness of child and adolescent mental health should be further promoted (from 30-40% of awareness in 2005 to 80% in 2015). The plan also emphasizes that relevant information on the prevention and screening of mental disorders be accessible and distributed by primary care physicians. The Developing Outline for Chinese women and Children in 2010 [79] also emphasized the importance of child mental health and that multiple forms of psychological counseling and treatment programs be provided to the public.

## Conclusions and future perspectives

Despite all the new laws and regulations, the dearth of child psychiatrists in China is expected to continue for some time. In order to address this problem, a new form of multilevel collaboration is being implemented. Pediatricians and primary care physicians are being trained in child psychiatry. Officials have also enlisted foreign psychotherapists to help train psychiatrists and increase awareness. China is now exploring all possible ways to enforce the multilevel collaboration to promote the physical and psychological wellbeing of children.

A growing need for international collaboration is also seen in this field. From the time of Dr. Guotai Tao, the founding father of Chinese child psychiatry, who received his training in the United States, more child psychiatrists are involved in education and training programs overseas. China is an active member of the Asian Society for Child and Adolescent Psychiatry and Allied Professions (ASCAPAP) and the International Association for Child and Adolescent Psychiatry and Allied Professions (IACAPAP). Hopefully, with the effort of the government, society and a strengthened international collaboration, a public mental health framework with appropriate policies and programs, to educate and advocate for change, and to provide systemic and targeted solutions can be achieved.

## Abbreviations

ABC: Autism behavior checklist
ASD: Autistic spectrum disorders
ADHD: Attention deficit hyperactivity Disorders
ASCAPAP: The Asian society for child and adolescent psychiatry and allied professions
CABS: Clancy autism behavior scale
CARS: Childhood autism rating scale
CBCL: Child behavior checklist
CCMD: Chinese classification of mental disorders
CHAT: Checklist for autism in toddlers
DSM: Diagnostic and statistical manual of mental disorder
IACAPAP: International association for child and adolescent psychiatry and allied professions
ICD: International classification of diseases
TCM: Traditional Chinese medicine
TD: Tourette disorder
